# Cochlear Implantation after Kidney Transplantation

**Published:** 2012-08-01

**Authors:** S. B. Hashemi, H. Bahrani Fard, Z. Zandifar

**Affiliations:** *Department of Otolaryngology and Head and Neck Surgery, Khalili Hospital, Shiraz University of Medical Science, Shiraz, Iran*

**Keywords:** Cochlear implantation, Kidney transplantation

## Abstract

Patients with chronic renal failure may develop sensorineural hearing loss. Cochlear implantation has rarely done after organ transplantation. Herein, we report on a 33-year-old kidney transplantation recipient who underwent cochlear implantation for her progressive sensorineural hearing loss in Khalili Hospital Cochlear Implant Center, affiliated to Shiraz University of Medical Sciences. The implantation was done successfully with no complications. Cochlear implantation may be an appropriate therapeutic option for sensorineural hearing loss caused by chronic renal failure.

## INTRODUCTION

Cochlear implantation has revolutionized the treatment of profound hearing loss. This procedure is relatively safe and it becomes the treatment of choice for both children and adults. Most of the patients reported improved quality of life, speech recognition and communication. Recently, patients with chronic diseases like chronic renal failure with hearing impairment, have also considered for cochlear implantation. Chronic kidney disease may result in high frequency sensorineural hearing loss (SNHL). The probable causes include uremia [1], congenital nephropathy [2, 3], infection, electrolyte imbalance [4] and administration of ototoxic drugs [3, 5]. Cochlear implantation may help these patients.

## CASE REPORT

A 15-year-old Iranian girl developed chronic renal failure due to post-streptococal glomerulonephritis. Although she was on maintenance hemodialysis for seven months, she developed end-stage renal disease and underwent living non-related kidney transplant in 1992. One month later, she was treated for pleural effusion and pneumonia and received the ototoxic drug, amikacin for several weeks and developed bilateral progressive SNHL confirmed by pure tone audiometry and auditory brainstem response audiometry. She was then referred to our center and received a powerful hearing aid which was not successful to increase her speech awareness threshold. Therefore, she was considered for cochlear implantation. Before performing the procedure, multidetector high resolution computed tomography of temporal bone revealed normal anatomy of the temporal bone including mastoid, middle ear, inner ear and internal auditory canal with no evidence of mastoiditis or pathologic findings. The cochlear turns in both sides appeared normal with normal semicircular canals. After evaluation, the right ear was selected for the implantation. An advance bionics prosthesis (HiRes 90 K) was used and the active electrode was inserted fully in an appropriate position in the year 2011. After the operation, the patient was hospitalized in the ENT ward for 24 hours and discharged in good condition with antibiotics. She continued her routine immunosuppressant drugs till now without any signs of wound infection or other complications.

## DISCUSSION

Hearing aids are the principle means of auditory rehabilitation for patients with SNHL. Hearing aids also have important roles in the management of conductive hearing loss. Implantable hearing aid has several advantages for patients with hearing loss such as increasing gain and dynamic range, reducing feedback, reducing maintenance, improving appearance and being free to occlude one of the ear canals [6]. Cochlear implant is a safe procedure, which is used for severe to profound SNHL, with low complication rate.

Hearing loss is one of the most common debilitating conditions in patients who begin renal replacement therapy. The incidence of hearing loss in children is 8.6% [3]. The effect of kidney transplantation on hearing function is controversial [3]. Another report mentioned that although kidney transplantation might improve the hearing function at first, in the long term it worsens the hearing capacity [7]. The underlying cause for this process is not still clear, but it might be attributed to vascular changes in the inner ear due to hyperlipidemia induced by steroid therapy, treatment with ototoxic drugs or recurrence of renal disease [8]. Since SNHL is a common complication of chronic renal failure, cochlear implant is performed after kidney transplantation. If the cochlear transplantation is done before organ transplantation or within the six months of organ transplantation, the postoperative complications would increase [5, 9].

For cochlear implantation, immunosuppressive drugs can be continued preoperatively without dose adjustments [2, 5, 9]. Furthermore, some studies recommended stress dose of steroid before cochlear implantation [9], because patients had been treated with long-term steroid and this method would decrease postoperative complication rates. They also showed that preoperative prophylactic antibiotic therapy would decrease postoperative complication rates [2, 5, 9].

We concluded that cochlear implant may help properly selected renal transplant recipients with SNHL.
